# The N6-Methyladenosine Regulator *ALKBH5* Mediated Stromal Cell–Macrophage Interaction via VEGF Signaling to Promote Recurrent Spontaneous Abortion: A Bioinformatic and In Vitro Study

**DOI:** 10.3390/ijms232415819

**Published:** 2022-12-13

**Authors:** Yongbo Zhao, Jiani Sun, Liping Jin

**Affiliations:** 1 Shanghai Key Laboratory of Maternal Fetal Medicine, Shanghai First Maternity and Infant Hospital, School of Medicine, Tongji University, Shanghai 200092, China; 2 Shanghai Key Laboratory of Maternal Fetal Medicine, Shanghai Institute of Maternal-Fetal Medicine and Gynecologic Oncology, Shanghai First Maternity and Infant Hospital, School of Medicine, Tongji University, Shanghai 200092, China

**Keywords:** N6-methyladenosine, recurrent spontaneous abortion, ALKBH5, macrophage, VEGF

## Abstract

Successful conception requires the synchrony of multiple systems and organs. Dysregulation of stromal cell–immune cell interactions has been proposed to be associated with recurrent spontaneous abortion. However, the mechanism of this regulation has not been well elucidated. N6-methyladenosine is one of the most common RNA modifications, and is involved in many pathological processes. Our group has demonstrated that abnormal patterns of m6A modification inhibit trophoblast invasion and contribute to adverse pregnancy outcomes. The association between m6A regulators and stromal cell–immune cell interactions is unclear. We obtained RNA-seq profiles from a GEO dataset and identified differentially expressed m6A regulators between healthy controls and patients with a recurrent spontaneous abortion history. ROC curves, functional enrichment and subclassification analysis were applied to elucidate the role of m6A regulators in pregnancy. We verified the expression of m6A regulators and constructed an overexpression cell line in a coculture system to reveal *ALKBH5* function in stromal cell–macrophage interactions. We identified 11 differentially expressed m6A regulators between healthy controls and patients with a recurrent spontaneous abortion history. Then, we identified the correlation between “eraser” genes and “writer” genes. We tested the predictive abilities of the 11 m6A regulators based on another dataset and verified their expression in primary human endometrial stromal cells. We then subclassified three distinct patterns using the 11 genes and visualized genes related to immune infiltration and macrophage function in each cluster. *ALKBH5* was proven to be correlated with recurrent spontaneous abortion. To verify the role of *ALKBH5* in RSA, we constructed an *ALKBH5*-overexpression cell line. Finally, we cocultured the overexpression cell line with THP-1 cells. A decrease in M2 differentiation was observed, and this bias could be attributed to the hyposecretion of VEGF in stromal cells. N6-methyladenosine regulators play a pivotal role in stromal cell–immune cell interactions at the maternal–fetal interface. Overexpression of the m6A “eraser” gene *ALKBH5* in stromal cells resulted in the hyposecretion of VEGF. Dysregulation of VEGF might impair macrophage recruitment and M2 differentiation, which could be the potential cause of recurrent spontaneous abortion.

## 1. Introduction

Recurrent spontaneous abortion (RSA) is a distressing pregnancy disorder affecting ~2.5% of women who are trying to conceive. Successful conception requires the coordination of multiple systems and organs, and maternal immunological and endometrial synchrony is one of them [1]. Endometrial stromal cells (ESCs) are key components of the endometrium. During the window of implantation, ESCs surround and establish a direct interaction with the embryo. As the embryo develops, ESCs decidualize, and decidual stromal cells (DSCs) produce numerous factors to mediate the activity of endothelial vascular cells, epithelial cells and immune cells [2,3,4]. Endometrial immune dysfunction has been proposed to be associated with RSA. Among the immunocyte profiles, macrophages, with their flexible plasticity, play a key role in maintaining conception [5]. Although the dominance of macrophage differentiation status continues to shift throughout the process of pregnancy, disrupting the balance may impair the maternal–fetal interface immune microenvironment and result in embryonic loss [6]. ESC/DSC-derived cytokines are involved in macrophage polarization [3]. However, the mechanism of ESC/DSC–macrophage interactions is still unclear.

N6-methyladenosine (m6A) has been identified as one of the most common and abundant RNA modifications. m6A modification is catalyzed by corresponding enzymes called “writers,” “erasers” and “readers”, which can install, remove and recognize, respectively. More than 7000 coding and 300 noncoding RNAs are estimated to contain m6A, and they affect mRNA splicing, stabilization and translation [7]. Aberrancies of m6A modification are associated with a variety of diseases, including female fertility, follicle development, oocyte maturation and RSA. Recent studies found that abnormal patterns of m6A modification inhibited trophoblast invasion and impaired immunotolerance and immune-cell infiltration at the maternal–fetal interface [8,9]. However, there is limited evidence of the role of m6A modification in RSA-related stromal cell–immune cell interactions.

In this study, we comprehensively evaluated the expression level of m6A regulators in healthy controls and RSA patients based on the GSE165004 dataset from the Gene Expression Omnibus (GEO) database. We then identified the subtype classification based on the differentially expressed m6A regulators. A cluster was characterized by high expression of the m6A “eraser” gene *ALKBH5* and impaired macrophage differentiation. We hypothesized that this cluster might be linked to RSA. The findings of our study could support a new theory for stromal cell–macrophage interactions and provide guidance for novel immune therapy for RSA patients.

## 2. Results

### 2.1. Landscape of the m6A Regulators in RSA

**Figure 1:**
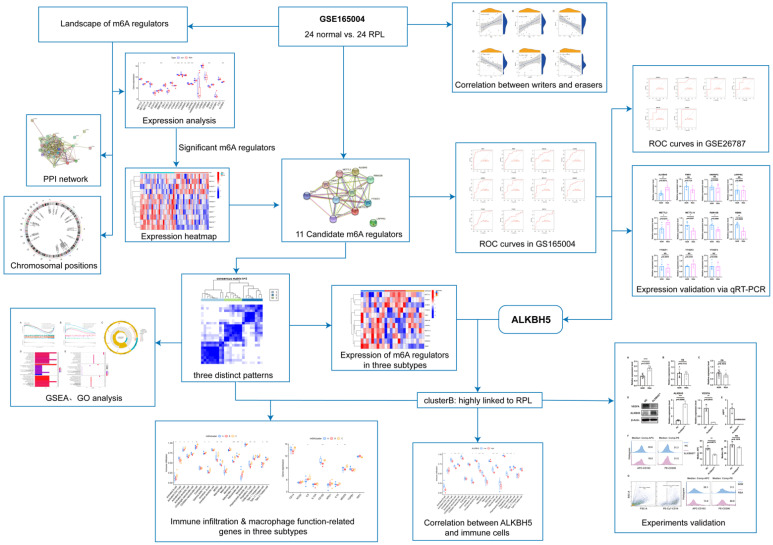
Workflow diagram illustrating the design and processes of our study.

**Figure 2:**
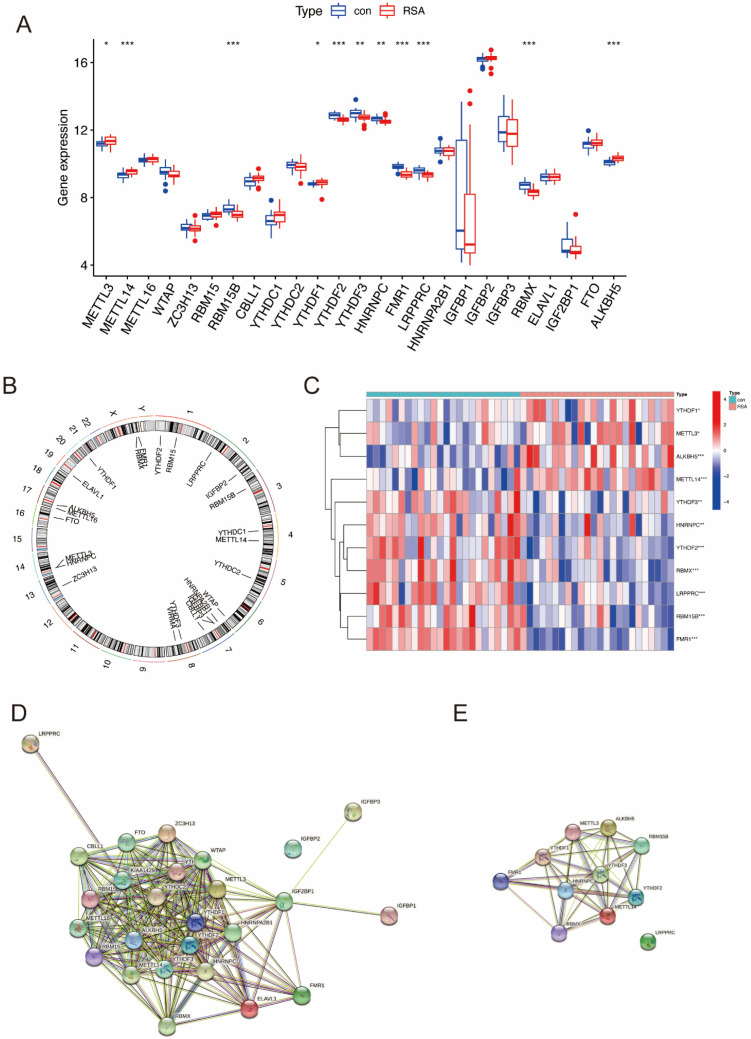
The landscape of the 25 m6A regulators in RSA. (**A**) Differential expression levels of the 25 m6A regulators between normal group and RSA patients. (**B**) Chromosomal positions of the 25 m6A regulators. (**C**) Expression heatmap of 11 significant m6A regulators. (**D**) PPI network of the 25 m6A regulators. (**E**) PPI network of the 11 significant genes. *: *p* < 0.05, **: *p* < 0.01, ***: *p* < 0.001.

The workflow (Figure 1) was plotted to show the process of our study. A total of 25 m6A regulators between healthy controls and RSA patients were extracted utilizing the “limma” package in R (Figure 2A). The chromosomal position was visualized using the “RCircos” package (Figure 2B). Among the 25 m6A regulators, a total of 11 differentially expressed m6A regulators were visualized in a heatmap (Figure 2C). Protein-protein interactions between the 25 m6A regulators and the 11 differentially expressed m6A regulators are shown in Figure 2D,E, respectively.

### 2.2. Identification of the Correlation between Writers and Erasers in RSA

**Figure 3:**
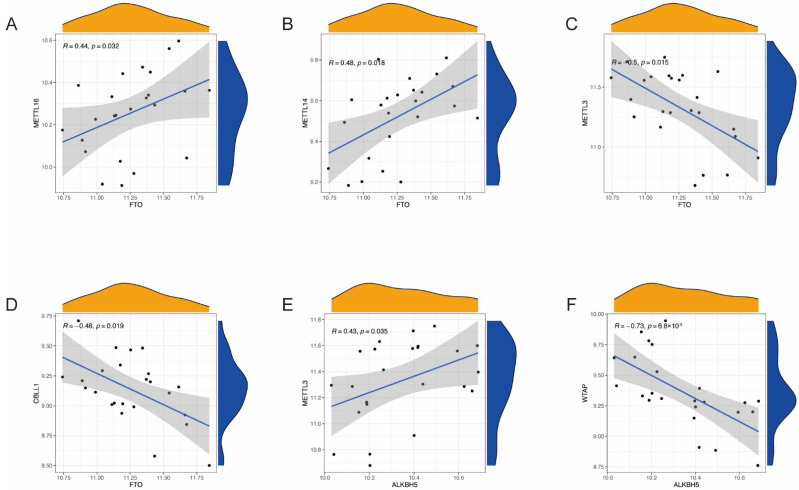
Correlation between writers and erasers in RSA. (**A**–**D**) Writer genes: *METTL16*, *METTL14*, *METTL3*, and *CBLL1*; eraser gene: *FTO*. (**E**,**F**) Writer genes: *METTL3* and *WTAP*; eraser gene: *ALKBH5*.

To explore whether low expression levels of eraser genes (*ALKBH5* and *FTO*) match higher expression levels of writer genes (*RBM15B*, *RBM15*, *METTL3*, *METTL14*, *WTAP*, *CBLL1*, and *ZC3H13*) in RSA, we utilized linear regression analysis to construct the correlation between writers and erasers. We found that two writer genes were significantly correlated with the eraser gene *ALKBH5*, and four writer genes were significantly correlated with the eraser gene *FTO* (Figure 3).

### 2.3. Identification and Validation of the Predictive Ability of 11 m6A Regulators

**Figure 4:**
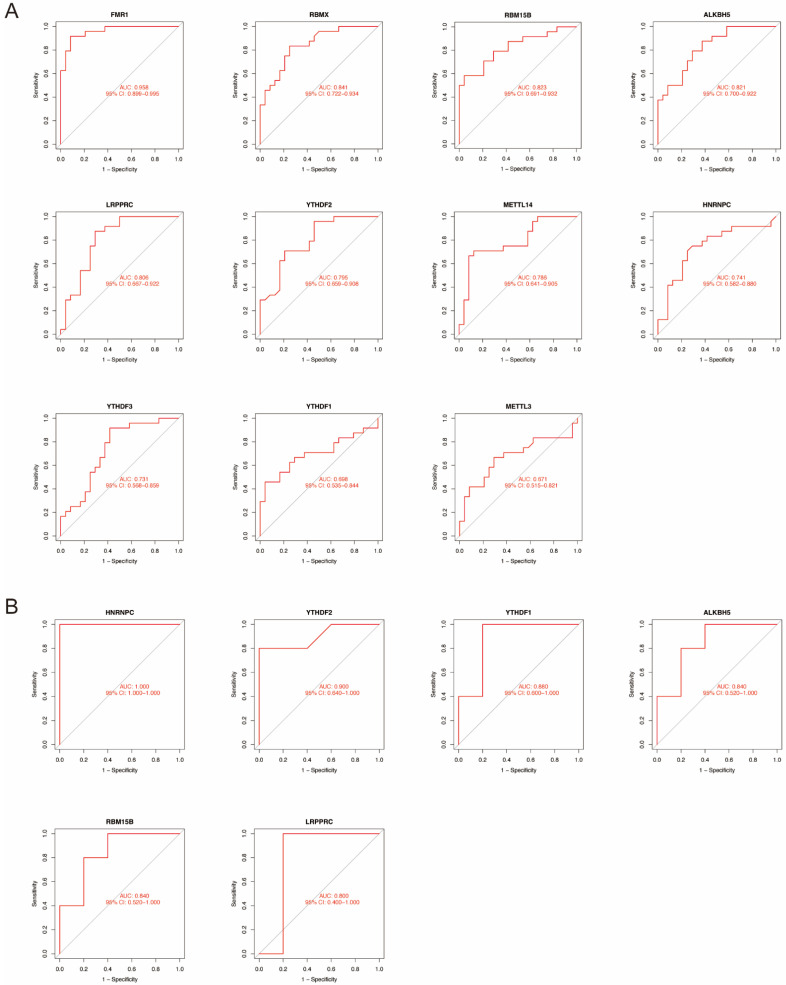
Construction of ROC curves. (**A**) ROC curves of significant genes based on GSE165004. (**B**) ROC curves based on GSE26787.

To test the diagnostic value of the candidate m6A regulators, we constructed ROC curves based on GSE165004. The area under the ROC curve visualizes the predictive ability of each candidate gene. The AUCs of all 11 m6A regulators were greater than 0.65, and the AUCs of 5 m6A regulators (*FMR1*, *RBMX*, *RBM15B*, *ALKBH5*, and *LRPPRC*) were greater than 0.8 (Figure 4A). To further test the predictive accuracy of the candidate genes, we constructed ROC curves based on GSE26787. Those with AUCs greater than 0.8 are shown in Figure 4B.

**Figure 5:**
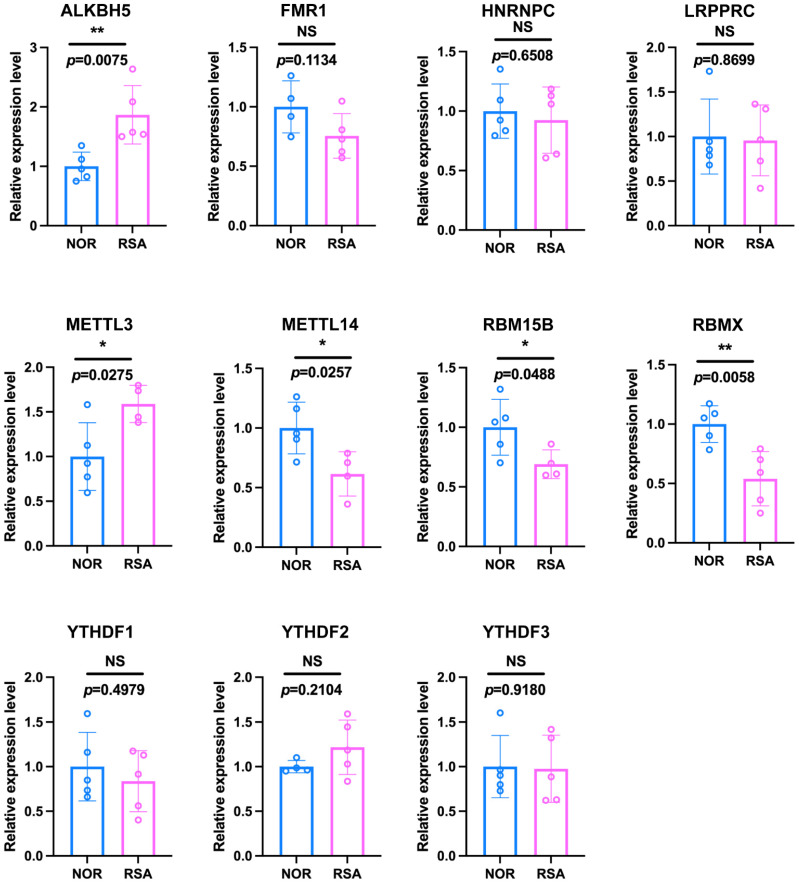
mRNA expression levels of significant m6A regulators in primary human ESCs. *: *p* < 0.05, **: *p* < 0.01, NS: Non-significance.

We used ESCs, the most abundant component of endometrial tissue, to validate the expression of m6A regulators. Consistent with the bioinformatic analysis, *ALKBH5* and *METTL3* were upregulated, while *METTL14*, *RBM15B* and *RBMX* were downregulated (Figure 5).

### 2.4. Identification of Three Distinct Patterns in RSA and Functional Enrichment Analysis

**Figure 6:**
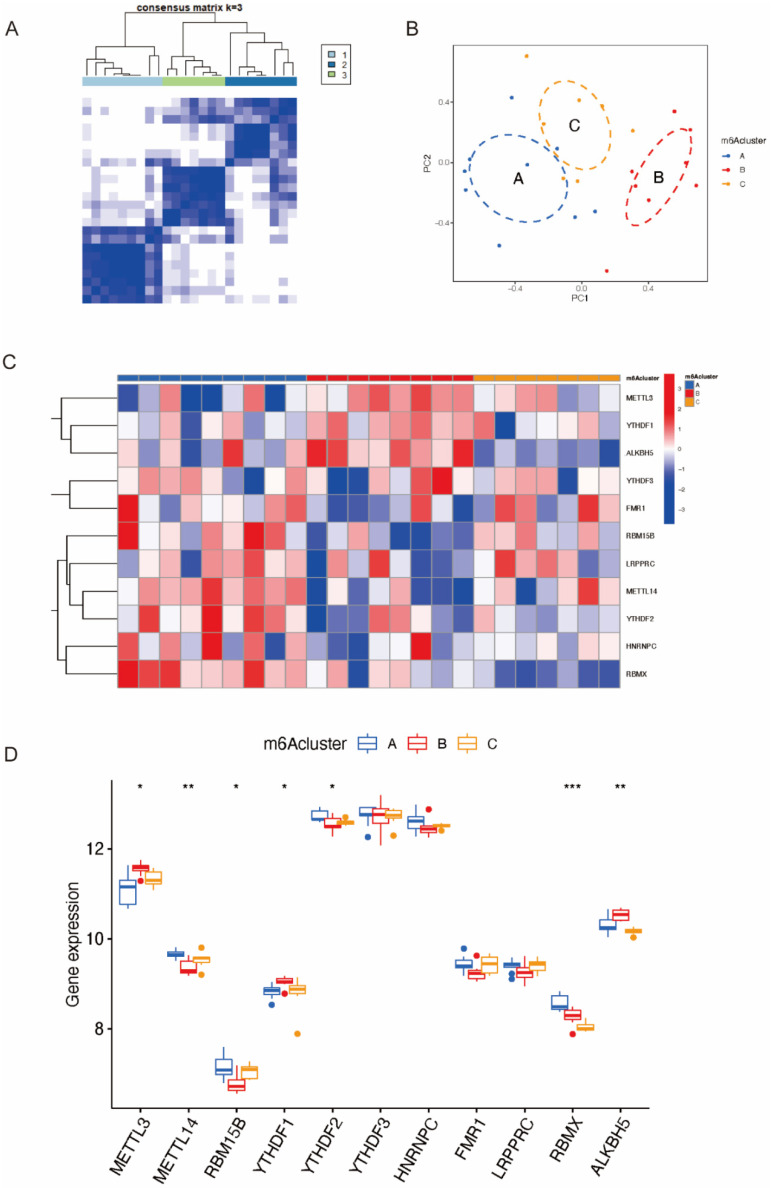
Identification of three distinct m6A patterns. (**A**) Three distinct m6A patterns based on significant genes. (**B**) Principal component analysis. (**C**) Expression heatmap of 11 candidate genes in three subtypes. (**D**) Differential expression histogram of 11 significant genes in three subtypes. *: *p* < 0.05, **: *p* < 0.01, ***: *p* < 0.001, NS: Non-significance.

Utilizing the “ConsensusClustePlus” package, we distinguished three distinct m6A patterns based on the 11 candidate m6A regulators via an unsupervised clustering method (Figure 6A,B). According to the PCA, the three patterns were distinguished clearly with a stable distribution of samples (Figure 6C). The expression of significant m6A regulators in the three clusters is shown in Figure 6D, from which we found that *ALKBH5* was overexpressed in Cluster B. Additionally, the process of constructing the consensus clustering is shown in Figure S1. We tried establishing the model with a consensus matrix from k = 2 to k = 9 (Figure S1A), and the consensus CDF curve and the relative change in the area under the CDF curve are shown in Figure S1B,C, respectively.

**Figure 7:**
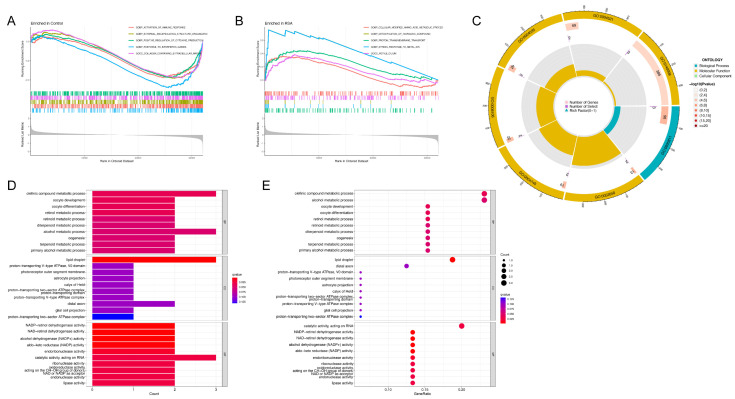
GSEA and functional enrichment of DEGs. (**A**) Enrichment analysis in the control group via gene set enrichment analysis. (**B**) Enrichment analysis in the RSA group via gene set enrichment analysis. (**C**) GO functional enrichment analysis based on DEGs among three subtypes in the circle chart. (**D**) GO functional enrichment analysis based on DEGs among three subtypes in the barplot chart. (**E**) GO functional enrichment analysis based on DEGs among three subtypes in the bubble chart.

The differentially expressed genes between the normal control group and the RSA group were extracted and subjected to GSEA. The GSEA results (Figure 7A,B) show that the DEGs were mainly enriched in the activation of immune response in the control group and mainly involved in the cellular modified amino acid metabolic process in the RSA group. In addition, the DEGs among the three distinct m6A subtypes were also extracted and subjected to gene ontology enrichment analysis (Figure 7C–E). The results of GO analysis (Table 1) showed that the DEGs were mainly enriched in BPs, consisting of the olefinic compound metabolic process, the alcohol metabolic process and so on. In terms of CCs, the DEGs were mainly involved in lipid droplets and distal axons; in terms of MFs, they were enriched in catalytic activity acting on RNA and metabolism of NAD.

### 2.5. Genes Related to Immune Infiltration and Macrophage Function Related in Different m6A Patterns

**Figure 8:**
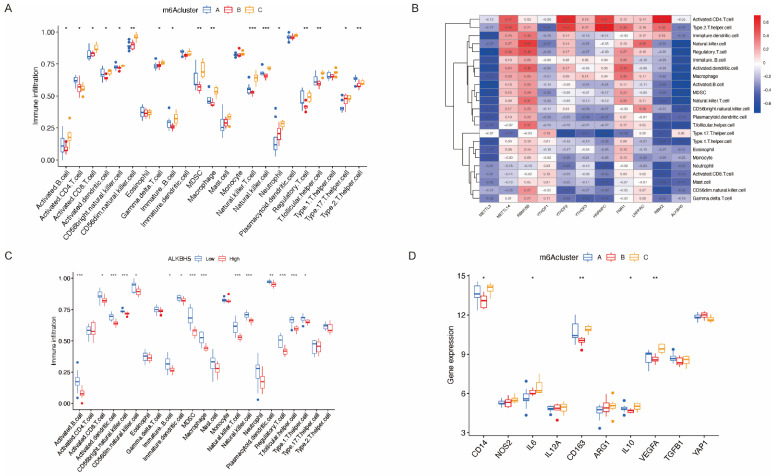
Analysis of genes related to immune infiltration and macrophage function. (**A**) The relationship between three subtypes and the infiltration of 23 immune cells. (**B**) Correlation between significant genes and 23 kinds of immune cells. (**C**) Variation and difference in the immune-cell infiltration between low and high ALKBH5 expression groups. (**D**) Analysis of macrophage-function-related genes in three subtypes. *: *p* < 0.05, **: *p* < 0.01, ***: *p* < 0.001.

ssGSEA was applied to calculate the abundance of immune cells in Clusters A–C (Figure 8A) and evaluate the correlation between 11 significant m6A regulators and immune cells (Figure 8B). We noticed that *ALKBH5*, the only differentially expressed m6A eraser gene in the dataset, was the most relevant m6A regulator with macrophage function. The analysis of *ALKBH5* and immune cells (Figure 8C) indicated that upregulation of *ALKBH5* in Cluster B might result in macrophage dysfunction. We also investigated the correlation between macrophage-function-related genes and m6A patterns (Figure 8D). The upregulation of IL6 and downregulation of CD163 and IL10 in Cluster B indicated that anti-inflammatory M2 differentiation was impaired, which could be a possible cause of RSA.

### 2.6. Overexpression of ALKBH5 in ESCs Affected Macrophage Differentiation via VEGF Secretion

**Figure 9:**
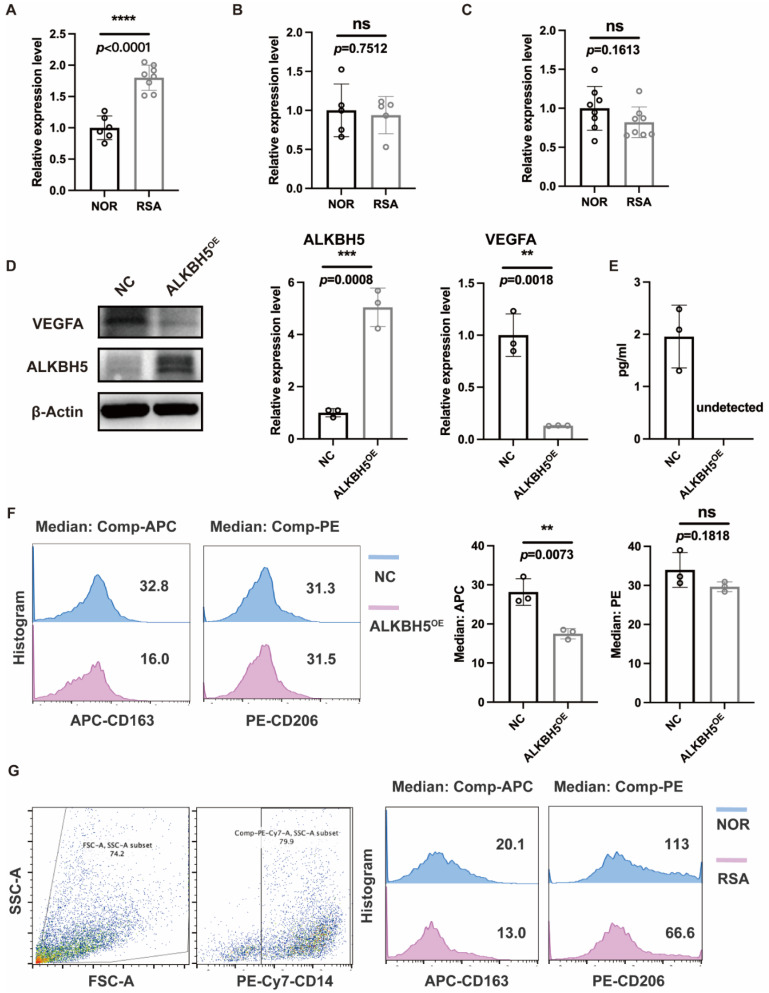
ALKBH5 altered macrophage differentiation status via VEGF secretion**.** (**A**) mRNA expression level of ALKBH5 in primary human DSCs. (**B**) mRNA expression level of ALKBH5 in primary human decidual NK cells. (**C**) mRNA expression level of ALKBH5 in primary human decidual macrophage. (**D**) Protein level of ALKBH5 and VEGFA in ALKBH5-overexpression ESC line. (**E**) VEGF concentration of ALKBH5-overexpression ESC line supernatant. (**F**) CD163 and CD206 median fluorescence intensity of THP-1 after co-culturing with ALKBH5-overexpression ESC line. (**G**) CD163 and CD206 median fluorescence intensity of primary human decidual macrophage. **: *p* < 0.01, ***: *p* < 0.001, ****: *p* < 0.0001, NS: Non-significance.

Due to the limited number of immune cells in endometrial tissue, we verified macrophage function in the decidua. ALKBH5 was also highly expressed in the DSCs (Figure 9A) of RSA patients, but not in decidual NK cells (Figure 9B) or decidual macrophages (Figure 9C). Therefore, we hypothesized that the dysfunction of macrophages was partially attributed to the overexpression of ALKBH5 in DSCs. We constructed an ALKBH5-overexpressing human ESC line to better understand its function. Based on the results shown in Figure 8D, we noticed that in the ALKBH5^high^ cluster, the expression of VEGF was relatively low. Therefore, we measured both the intracellular and supernatant levels and confirmed the insufficient secretion of VEGF in the ALKBH5-overexpressing human ESC line (Figure 9D,E). Considering that VEGF signaling is an important mediator of macrophages, we established a coculture system of ALKBH5-overexpressing human ESC lines and THP-1 cells, and the M2 differentiation status of THP-1 cells was impaired in the ALKBH5-overexpression group (Figure 9F). This CD163^low^ CD206^low^ decidual macrophage was observed in some of the RSA patients (Figure 9G).

## 3. Discussion

Recurrent spontaneous abortion is prevalent but underestimated. A Japanese group found that the live birth rates of pregnant women with RSA history were similar in 2011–2018 to those who returned in 1994–2010 (63.8% vs. 67.8%) [10]. This clearly indicated that new ideas for RSA therapy were needed. Established evidence has shown that m6A regulators are involved in pregnancy maintenance [11]. However, the significance of m6A modification in RSA-related stromal cell–immune cell interactions remains unknown. In our study, we aimed to identify distinctive m6A regulators in RSA and reveal the mechanism of m6A regulator mediation of stromal cell–immune cell interactions.

We first identified 11 significant m6A regulators among 25 m6A regulators through differential expression analysis of normal and RSA patients. We then constructed ROC curves of the 11 candidate genes to demonstrate their predictive abilities for RSA. To test the predictive accuracy, we plotted the ROC curves of another RSA dataset and validated the expression of significant genes via qRT-PCR. *ALKBH5*, whose AUCs were greater than 0.8 in both datasets, was proven to be significantly overexpressed in RSA samples, indicating that *ALKBH5* might be a potential biomarker for predicting RSA. Based on the 11 candidate genes, we further classified three distinct patterns, among which *ALKBH5* was significantly upregulated in Cluster B. Therefore, we hypothesized that Cluster B was highly linked to RSA. Subsequently, we performed immune infiltration analysis and identified macrophage-function-related genes. ssGSEA indicated that *ALKBH5* might jeopardize macrophage differentiation to promote RSA, and the mechanism might be the ESC-derived hyposecretion of VEGF. qRT-PCR, Western blotting, flow cytometry and ELISA experiments were subsequently performed to validate the bioinformatic results.

*ALKBH5*, a kind of primary m6A demethylase involved in alkylated DNA repair, is involved in different biological processes, including invasion, ossification, metastasis and proliferation [12,13]. The dysregulation of *ALKBH5* plays a crucial role in various kinds of diseases by regulating or interacting with other genes [14]. *ALKBH5* was reported to play an essential role in trophoblast invasion at the maternal–fetal interface [8,15], and was proven to be significantly overexpressed in infertile women [11].

Macrophages are involved in all stages of pregnancy, from embryonic implantation and placental formation to final delivery [16,17,18,19]. The precise macrophage differentiation status has not achieved consensus. However, this differentiation balance is carefully maintained, and the dysfunction of polarization is associated with adverse pregnancy outcomes. A decrease in CD163 and impaired M2 polarization activate macrophage apoptosis, contributing to the pathological process of RSA [20]. During the growing phase of pregnancy, the predominance of macrophage immune status was anti-inflammatory. Several studies revealed that as pregnancy advanced, M1 markers such as TLR9, IL1B, IL12RB2, CD48 and FGR were silenced, whereas the M2 surface marker CD206 and cytokines such as IL10, CCL13, CCL14 and IDO were upregulated [21,22]. Moreover, fetal-derived macrophages, Hofbauer cells, showed M2 characteristics with high expression of IL10, TGF-β, DC-SIGN, CD206 and CD163 [23]. VEGF is one of the pivotal growth factors produced in the decidua, and regulates the local immune microenvironment. VEGF in the decidua might contribute to macrophage recruitment and M2 polarization [24].

In our study, due to the limited number of immune cells in endometrial tissue, we focused on the interaction of DSCs and decidual immune cells. We recognized that decidualization is a complicated process and that the expression levels of many genes were altered. To minimize this effect, we confirmed ALKBH5 expression in primary human DSCs, which was consistent with the result in primary human ESCs. Additionally, excellent work has been published about ALKBH5 function in immune cells [25]. To rule out the potential mediation of ALKBH5 in decidual immune cells, we verified ALKBH5 expression in both decidual NK cells and macrophages, the top two immune-cell components in the decidua. We found that ALKBH5 was mainly differentially expressed in ESCs and DSCs, so we focused on stromal cell–immune cell mediation. In addition, VEGF is one of the major factors secreted by stromal cells, and evidence of its role in macrophage differentiation has been validated [26]. In our study, we analyzed and identified the crosslink between the ALKBH5^high^ cluster and VEGF^low^ cluster. This result provides us with evidence that the phenomenon of ALKBH5-related macrophage dysfunction might be correlated with insufficient secretion of VEGF by endometrial stromal cells. One of the drawbacks of our study was that we were unable to identify the downstream molecules of ALKBH5 and the unidentified molecules that might regulate VEGF secretion. Further study should be performed to answer these scientific questions. Additionally, there was another limitation in our research. Though we chose a relatively reliable consensus matrix, there was still some over-fitting in the consensus clustering due to the limited sample numbers. We plan to carry out further research with larger sample numbers in order to generate a better consensus-clustering model.

## 4. Materials and Methods

### 4.1. Data Acquisition and PPI Network Construction

The GSE165004 dataset containing 24 healthy controls and 24 RSA patients was obtained from the GEO database. We extracted a total of 25 m6A regulators from the dataset. Eleven were differentially expressed, consisting of three writers (*METTL3*, *METTL14*, *RBM15B*), one eraser (*ALKBH5*) and seven readers (*YTHDF1*, *YTHDF3*, *HNRNPC*, *YTHDF2*, *LRPPRC*, *FMR1*, *RBMX*). Linear regression analysis was used to detect the correlation between erasers and writers. The 25 m6A regulators and 11 differentially expressed regulators were utilized to construct the protein-protein interaction network using STRING V11.5 (https://string-db.org/ (accessed on 3 March 2022)) [27]. In addition, GSE26787 [28], containing 5 RSA patients and 5 healthy controls, was utilized as the validation dataset.

### 4.2. Construction of Receiver Operating Characteristic (ROC) Curves

We constructed ROC curves of the candidate m6A regulators to identify their predictive ability via the “pROC” package. The area under the ROC curve (AUC) illustrates the predictive accuracy of each m6A regulator. In addition, the predictive ability of the significant m6A regulators was validated using the GSE26787 dataset.

### 4.3. Classification of Three Distinct m6A Subtypes and Extraction of DEGs

Consensus clustering is a useful method to subclassify members. Based on the significant m6A regulators, we utilized consensus clustering to classify the RSA patients into subtypes via the “ConsensusClustePlus” package [29]. Principal component analysis (PCA) was applied to distinguish and identify m6A patterns. Differentially expressed genes (DEGs) among the distinct m6A patterns, and between the control and RSA groups, were extracted using the “limma” package [30] with the criterion of *p* < 0.05.

### 4.4. Functional Enrichment Analysis of DEGs

Differentially expressed genes between the control and RSA groups were extracted for gene set enrichment analysis (GSEA) to reveal the potential mechanism of RSA. We downloaded and utilized “c2.cp.kegg.v7.4.symbols.gmt” as the reference gene set with the criterion of a false discovery rate (FDR) < 0.05 and adjusted *p* < 0.05.

We obtained 20 differentially expressed genes from the cluster differential expression analysis (Table S1), and the 20 DEGs among three distinct m6A patterns were utilized for gene ontology (GO) functional enrichment analysis via the “clusterProfiler” package [31]. Three aspects of the GO biological processes were visualized, the biological processes (BPs), cellular components (CCs) and molecular functions (MFs).

### 4.5. Single-Sample Gene Set Enrichment Analysis to Identify Immune-Cell Infiltration

The variety of immune cells plays an essential role in the mechanism of RSA. To identify the characteristics of immune cells in RSA samples, we applied single-sample gene set enrichment analysis (ssGSEA) [32]. The abundance of immune cells in RSA samples and the correlation between candidate m6A regulators and immune cell infiltration could be visualized through ssGSEA.

### 4.6. Cell Culture

Primary human ESCs were collected from uterine endometrial biopsies for benign gynecological disorders. Women undergoing hysterectomy provided informed consent and were anonymized during ESC isolation. This study was reviewed and approved by the Ethics Committee of Shanghai First Maternity and Infant Hospital. The isolation and purification procedure was performed as previously described [33], which supplied 98% pure ESCs. Both primary and immortalized human ESC lines were cultured in phenol red-free Dulbecco’s modified Eagle medium (DMEM)/Ham’s F12 with 10% fetal bovine serum (FBS), 100 U/mL penicillin and 100 μg/mL streptomycin.

Primary human decidual NK cells and macrophages were obtained from healthy pregnant women and patients with a diagnosed RSA history undergoing an elective abortion. All the RSA patients suffered a history of two or more spontaneous miscarriages. However, patients with uterine malformation, infections, chromosomal aberrations, or who underwent IVF treatment were excluded. The normal control group comprised women who chose to have elective abortions for nonmedical reasons. The isolation and purification procedure was performed as previously described [34]. The culture medium for primary decidual NK cells and the human macrophage cell line THP1 was RPMI 1640 with 10% fetal bovine serum, 100 U/mL penicillin and 100 μg/mL streptomycin. Primary decidual macrophages were cultured in DMEM with 10% fetal bovine serum, 100 U/mL penicillin and 100 μg/mL streptomycin. All cells were maintained in humidified conditions at 37 °C and 5% CO_2_.

### 4.7. Antibodies for Flow Cytometry (FCM)

To evaluate macrophage differentiation status, primary decidual macrophages and THP1 cells were stained with APC-conjugated anti-human CD163 antibody and PE-conjugated anti-human CD206 antibody for anti-inflammatory M2 bias. Macrophage purity was validated with PE-Cy7-CD14 (all from BioLegend, Beijing, China).

### 4.8. Construction of an ALKBH5-Overexpressing ESC Cell Line

We constructed an ALKBH5-overexpressing ESC cell line using the pCMV6-AN-DDK vector, and the protein level of ALKBH5 was confirmed by Western blotting analysis. The primers for the ALKBH5-overexpression plasmid were as follows: gatgacgataaggcgatcgcCATGGCGGCCGCCAGCGG; gagtgcggccgcttaacgcgtTCAGTGCCGCCGCATCTT. Gene transfection was performed using a Lipo3000 kit (Invitrogen, Shanghai, China) following the manufacturer’s protocol.

### 4.9. Enzyme-Linked Immunosorbent Assay

The VEGF concentration in the supernatant was measured using an enzyme-linked immunosorbent assay (ELISA) kit (MULTI SCIENCES, Hangzhou, China, #EK183-02).

### 4.10. Quantitative Real-Time PCR (qRT-PCR)

Total RNA was extracted from primary human ESCs. Lysates were prepared using RNAiso Plus (Takara, Osaka, Japan). One microgram of total RNA was reverse transcribed into cDNA utilizing a PrimeScript™ RT reagent kit (Takara, Osaka, Japan). The primer sequences of 11 significant m6A regulators are listed in Table 2.

### 4.11. Western Blot

RIPA (Beyotime Biotechnology, Haimen, China, #P0013B) supplemented with a protease inhibitor cocktail (Roche, Branford, CT, USA, #11697498001) and phosphatase inhibitor cocktail (Roche, Branford, CT, USA, #4906837001) was used for sample lysis. Proteins (15–30 µg) were loaded and separated by 10% SDS—PAGE and then transferred to polyvinylidene fluoride membranes. The membranes were incubated with the following primary antibodies at 4 °C overnight: ALKBH5 (1:1000; Abcam, Cambridge, UK), β-actin (1:5000; Cell Signaling Technology, Danvers, MA, USA) and VEGFA (1:1000, Abcam, Cambridge, UK).

The blots were further incubated with an HRP-conjugated goat anti-rabbit IgG secondary antibody (1:3000, Abmart, Shanghai, China) for 1 h at room temperature. Protein visualization was performed using an enhanced chemiluminescence solution (Merck Millipore, Darmstadt, Germany, #32106). The relative protein expression levels were analyzed by densitometry using ImageJ software. β-actin was used as a loading control.

### 4.12. Statistical Analysis

After collecting the data, R software (version 4.0.0) was used for the statistical analysis. The correlation between writers and erasers was explored utilizing linear regression analysis. All parametric analyses were based on two-tailed tests with significance set at *p* < 0.05. Differences between two groups were compared with Student’s *t*-test. Multiple groups were compared using Kruskal-Wallis tests.

## 5. Conclusions

Eleven significant m6A regulators between the control group and the RSA group were extracted and utilized to apply bioinformatic analysis consisting of subtype classification and functional enrichment. We identified and validated the upregulation of the m6A eraser ALKBH5 in ESCs and DSCs of RSA patients. After identifying three distinct m6A patterns, we focused on Cluster B, with a high expression level of ALKBH5 and insufficient macrophage infiltration. We recognized this cluster to be highly correlated with stromal cell–macrophage interactions. Experiments were conducted to validate the hypothesis; ALKBH5 overexpression in the ESC line caused the hyposecretion of VEGF and subsequently downregulated the macrophage M2 marker. Alteration of the M2 differentiation status potentially promoted RSA.

## Figures and Tables

**Table 1:** The enriched GO terms of differentially expressed genes.

ID	Category	GO Name	*p*. Value	Count	Gene ID
GO:0120254	BP	olefinic compound metabolic process	0.00014411	3	DHRS3/RDH10/CYP2J2
GO:0048599	BP	oocyte development	0.00043323	2	PLD6/ANG
GO:0009994	BP	oocyte differentiation	0.00051382	2	PLD6/ANG
GO:0042572	BP	retinol metabolic process	0.00053502	2	DHRS3/RDH10
GO:0005811	CC	lipid droplet	6.13 × 10^5^	3	DHRS3/RDH10/CES1
GO:0052650	MF	NADP-retinol dehydrogenase activity	4.83 × 10^5^	2	DHRS3/RDH10
GO:0004745	MF	NAD-retinol dehydrogenase activity	0.00010559	2	DHRS3/RDH10
GO:0008106	MF	alcohol dehydrogenase (NADP+) activity	0.00014244	2	DHRS3/RDH10
GO:0004033	MF	aldo-keto reductase (NADP) activity	0.00024952	2	DHRS3/RDH10
GO:0004521	MF	endoribonuclease activity	0.00141489	2	PLD6/ANG
GO:0140098	MF	catalytic activity, acting on RNA	0.00347205	3	PLD6/RAD54B/ANG
GO:0004540	MF	ribonuclease activity	0.00393461	2	PLD6/ANG
GO:0016616	MF	oxidoreductase activity, acting on the CH-OH group of donors, NAD or NADP as acceptor	0.00448163	2	DHRS3/RDH10
GO:0004519	MF	endonuclease activity	0.00484046	2	PLD6/ANG
GO:0016298	MF	lipase activity	0.00484046	2	PLD6/CES1
GO:0016614	MF	oxidoreductase activity, acting on CH-OH group of donors	0.00536449	2	DHRS3/RDH10
GO:0015271	MF	outward rectifier potassium channel activity	0.01056786	1	KCNK2
GO:0004518	MF	nuclease activity	0.01238517	2	PLD6/ANG
GO:0008191	MF	metalloendopeptidase inhibitor activity	0.01299175	1	RARRES1
GO:0022841	MF	potassium ion leak channel activity	0.01299175	1	KCNK2
GO:0008392	MF	arachidonic acid epoxygenase activity	0.01379848	1	CYP2J2
GO:0042625	MF	ATPase-coupled ion transmembrane transporter activity	0.01379848	1	ATP6V0E2
GO:0044769	MF	ATPase activity, coupled to transmembrane movement of ions, rotational mechanism	0.01379848	1	ATP6V0E2
GO:0046961	MF	proton-transporting ATPase activity, rotational mechanism	0.01379848	1	ATP6V0E2
GO:0022840	MF	leak channel activity	0.0154101	1	KCNK2
GO:0022842	MF	narrow pore channel activity	0.0154101	1	KCNK2
GO:0009678	MF	pyrophosphate hydrolysis-driven proton transmembrane transporter activity	0.01621498	1	ATP6V0E2
GO:0008391	MF	arachidonic acid monooxygenase activity	0.01701926	1	CYP2J2
GO:0016866	MF	intramolecular transferase activity	0.02183201	1	CYP2J2

**Table 2:** The primer sequences of 11 significant m6A regulators.

	Forward Primer Sequence (5′→3′)	Reverse Primer Sequence (5′→3′)
*YTHDF1*	ACCTGTCCAGCTATTACCCG	TGGTGAGGTATGGAATCGGAG
*METTL3*	CATTGCCCACTGATGCTGTG	AGGCTTTCTACCCCATCTTGA
*ALKBH5*	AGTTCCAGTTCAAGCCTATTCG	TGAGCACAGTCACGCTTCC
*METTL14*	GAACACAGAGCTTAAATCCCCA	TGTCAGCTAAACCTACATCCCTG
*YTHDF3*	GCTATCCACCTAGTTCTCTTGGG	ATGCCAGGCACCTTACTCAAA
*YTHDF2*	CCTTAGGTGGAGCCATGATTG	TCTGTGCTACCCAACTTCAGT
*RBMX*	CTTCAGGACCAGTTCGCAGTA	TCACGACCACTTGAGTAGAGAT
*LRPPRC*	GCTCATAGGATATGGGACACACT	CCAGGAAATCAGTTGGTGAGAAT
*RBM15B*	TGATTGGTCGCAACCCCATTA	CAGTGACGTGTTAGGTCCCAG
*FMR1*	TATGCAGCATGTGATGCAACT	TTGTGGCAGGTTTGTTGGGAT
*HNRNPC*	TCCTCCTCCTATTGCTCGGG	GTGTTTCCTGATACACGCTGA

## Data Availability

The datasets for this study can be found in the GEO database. Please see the https://www.ncbi.nlm.nih.gov/geo/ (accessed on 1 March 2022) for more details.

## Supplementary Materials

The following supporting information can be downloaded at: https://www.mdpi.com/article/10.3390/ijms232415819/s1.
