# Supplementary material for: The N6-Methyladenosine Regulator ALKBH5 Mediated Stromal Cell–Macrophage Interaction via VEGF Signaling to Promote Recurrent Spontaneous Abortion: A Bioinformatic and In Vitro Study
Source: Int J Mol Sci. 2022 Dec 13;23(24):15819. doi: 10.3390/ijms232415819 (PMC9785252; doi:10.3390/ijms232415819)
Supplement: Supplementary file 1 [file ijms-23-15819-s001.zip › ijms-2064767-supplementary.pdf]

Table S1. The differentially expressed genes among three distinct m6A patterns.

Differentially expressed genes	
1	PLD6
2	TMEM45B
3	ATP6V0E2
4	PHYHIPL
5	GLT1D1
6	SFN
7	RAD54B
8	XLOC_007085
9	PRR15L
10	DHRS3
11	C2CD4B
12	RDH10
13	ACP6
14	CES1
15	CYP2J2
16	LOC100505483
17	RARRES1
18	KCNK2
19	XLOC_l2_006937
20	ANG

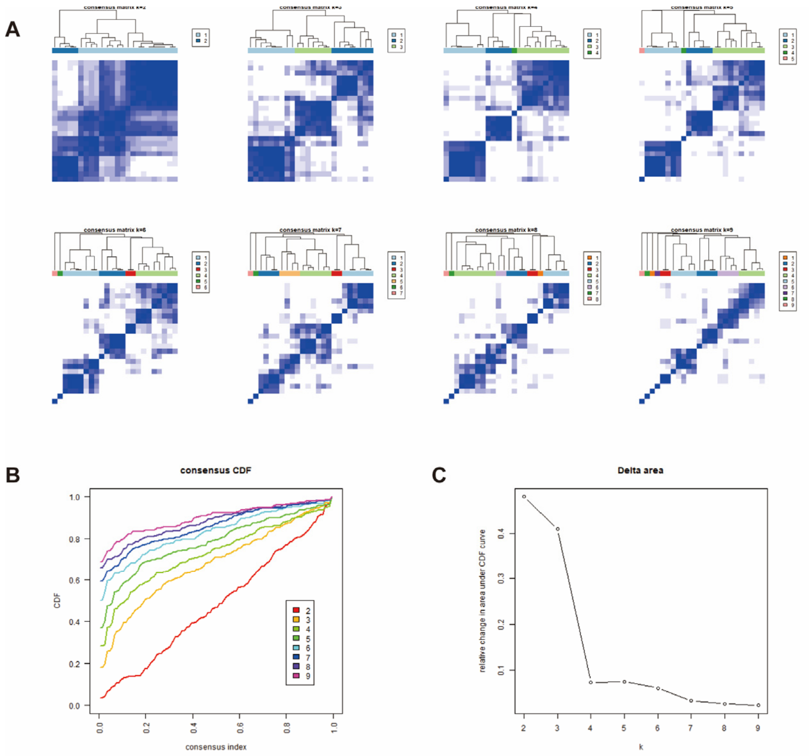

Figure S1. Construction of consensus clustering model. (A) Consensus clustering from consensus matrix k=2 to k=9. (B) The consensus CDF curve. (C) The relative change in area under CDF curve.
